# Serum metabolome changes in adult patients with severe dengue in the critical and recovery phases of dengue infection

**DOI:** 10.1371/journal.pntd.0006217

**Published:** 2018-01-24

**Authors:** Liang Cui, Junxiong Pang, Yie Hou Lee, Eng Eong Ooi, Choon Nam Ong, Yee Sin Leo, Steven R. Tannenbaum

**Affiliations:** 1 Infectious Diseases Interdisciplinary Research Group, Singapore-MIT Alliance for Research & Technology (SMART), Singapore, Singapore; 2 Communicable Disease Center, Institute of Infectious Diseases and Epidemiology, Tan Tock Seng Hospital, Singapore, Singapore; 3 Saw Swee Hock School of Public Health, National University of Singapore, Singapore, Singapore; 4 KK Research Centre, KK Women’s and Children’s Hospital, Singapore, Singapore; 5 Emerging Infectious Diseases Program, Duke-NUS Graduate Medical School, Singapore, Singapore; 6 NUS Environment Research Institute, National University of Singapore, Singapore, Singapore; 7 Departments of Biological Engineering and Chemistry, Massachusetts Institute of Technology, Cambridge, Massachusetts, United States of America; University of California, Berkeley, UNITED STATES

## Abstract

Dengue virus (DENV) is the most prevalent arbovirus leading to an estimated 100 million symptomatic dengue infections every year. DENV can cause a spectrum of clinical manifestations, ranging from mild dengue fever (DF) to more life threatening forms such as dengue hemorrhagic fever (DHF). The clinical symptoms of DHF become evident typically at the critical phase of infection (5–7 days after onset of fever), yet the mechanisms that trigger transition from DF to DHF are not well understood. We performed a mass spectrometry-based metabolomic profiling of sera from adult DF and DHF patients at the critical and recovery phases of infection. There were 29 differentially expressed metabolites identified between DF and DHF at the critical phase. These include bile acids, purines, acylcarnitines, phospholipids, and amino acids. Bile acids were observed up to 5 fold higher levels among DHF compared to DF patients and were significantly correlated to the higher levels of aspartate transaminase (AST) and alanine transaminase (ALT), suggestive of liver injury among DHF. Uric acid, the most abundant antioxidant in the blood, was observed to be 1.5 fold lower among DHF compared to DF patients. This could result in decreased capacity of endogenous antioxidant defense and elevated oxidative stress among DHF patients. In the recovery phase, the levels of eight metabolites were still significantly higher or lower among DHF patients, including chenodeoxyglycocholic acid, one of the bile acids observed at the critical phase. This indicates potential prolonged adverse impact on the liver due to DENV infection in DHF patients. Our study identified altered metabolic pathways linked to DHF in the critical and recovery phases of dengue infection and provided insights into the different host and DENV interactions between DF and DHF. The results advance our understanding on the mechanisms of DHF pathogenesis, alluding to possible novel therapeutic targets to dengue management.

## Author summary

Dengue is a re-emerging disease caused by dengue viruses (DENV) and half of the world’s population is now at risk of dengue infection. DENV can cause a spectrum of clinical manifestations ranging from mild dengue fever (DF) to the potentially lethal dengue hemorrhagic fever (DHF). The molecular mechanisms that trigger transition from DF to DHF are not well understood. To gain insights on the mechanisms of DHF pathogenesis, we performed metabolomic profiling of sera from adult DF and DHF patients at the critical phase of infection (5–7 days after onset of fever), the time point when the clinical symptoms of DHF become more evident. 29 differentially expressed metabolites between DF and DHF were identified, allowing us to discover a variety of metabolite pathways linked to DHF. They include bile acid biosynthesis, fatty acid *β*-oxidation, purine and pyrimidine metabolism, phospholipid catabolism, tryptophan and phenylalanine metabolism, and so on. The results advance our understanding on the mechanisms of DHF pathogenesis, and certain altered pathways being harnessed as therapeutic targets to alleviate DHF. These differentially expressed metabolites have the potential as biomarkers for disease monitoring and evaluation of therapeutic interventions.

## Introduction

Dengue is a re-emerging disease caused by four closely related serotypes of dengue viruses (DENV) and it is endemic in the tropical and sub-tropical regions of the world. An estimated 100 million symptomatic dengue infections occur annually, with over half of the world’s population living at the risk of infection [1]. Infection with DENV can cause a spectrum of clinical manifestations ranging from mild dengue fever (DF) to the potentially lethal dengue hemorrhagic fever (DHF) and dengue shock syndrome (DSS), which are characterized by abnormal hemostasis, vascular leakage and liver damage [2]. As yet, there is no specific treatment for dengue and the management of DHF patients is primarily supportive. Furthermore, the lack of an appropriate animal model poses great challenges in the study of dengue pathogenesis.

Dengue is a dynamic disease. After the incubation period, the illness begins abruptly and is followed by the three phases—febrile (day 0–4 post onset of fever), critical (day 5–7 post onset of fever) and recovery (day 21–28 post onset of fever). DF patients would recover uneventfully after 5–7 days of acute illness, but for DHF patients, the initial febrile period is followed by a rapid onset of vascular leakage, thrombocytopenia and hemorrhage at the critical phase. The continual loss of intravascular volume from plasma leakage can rapidly lead to hypotension and circulatory failure in DHF patients, which can result in death. The pathological difference between DF and DHF suggest differential virus-host interaction in the susceptibility to the disease, and both viral and host immune factors are likely involved. Although numerous efforts have been made in the last decade [3–6], the mechanisms that trigger transition from mild DF to more life threatening DHF at the critical phase are not fully understood, hampering the design of effective treatments for DHF.

Metabolomics is a rapidly emerging field in systems biology that refers to the global investigation of metabolite pool in biological systems in response to biological stimuli or perturbations [7]. Metabolites are the end products of cellular regulatory processes and form a direct link between molecular changes and phenotypes, and by providing a snapshot of an organism’s metabolic status, metabolomics holds the promise of finding metabolites or metabolic pathways related to disease processes [8,9]. It has been applied to infectious diseases to study host-pathogen interactions, including DENV infections [10–13]. In our previous study, we investigated serum metabolome difference between adult DF and DHF patients enrolled from a prospective dengue study at early febrile phase of DENV infection and found potential marker metabolite to predict DHF at early phase of the infection [14]. In the present study, metabolomics investigation was conducted on the same patient cohort at both the critical phase and recovery phases, with an aim to study the potential mechanisms that may contribute to the transition from DF to DHF at the critical phase of DENV infection. Interestingly, the majority of identified differentially expressed metabolites between DF and DHF in the febrile phase were different from those identified in the critical phase, showing that metabolomics study could capture the distinct molecular changes in DF and DHF patients at different phases of DENV infection. In the recovery phase, the levels of a few metabolites were still significantly altered in DHF patients, indicating prolonged effects of DENV infection on DHF patients. Our results identified metabolic pathways linked to dengue progression and provided insights on the mechanisms of DHF pathogenesis.

## Methods

### Ethics statement

Enrollment of all eligible individuals was based on written informed consent and the collected samples were anonymized. The protocols were approved by the Domain Specific Review Board of the National Healthcare Group, Singapore (DSRB/E/2009/432).

### Patient enrollment

The details of patient recruitment, sample collection and the study protocols of the study have been described earlier [14]. Briefly, the study cohort of dengue patients were recruited from the Prospective Adult Dengue Study (PADS), which is a cohort study of acute febrile adults at a tertiary care center, Communicable Diseases Center, Tan Tock Seng Hospital, Singapore. Adult patients (≥ 18 years) presenting with acute onset of fever (≥ 37.5°C) without rhinitis or other clinical alternatives were included in the study (Febrile stage, < 96 hours post onset of fever; Critical, Day 5–7, Convalescence, Day 21–28). Venous blood samples were collected, aliquoted and frozen at -80°C for hematological, virological and serological analysis. The study design, hypotheses, patient characteristics, assay methods, statistical methods and modeling methods were reported as per REMARK which is important for generalizability [15]. DF and DHF patients were classified according to the WHO 1997 and 2009 dengue guidelines [2,16]. To fulfill the case definition of DHF, all four of the following criteria must be present, namely: fever or history of fever, hemorrhagic tendencies, thrombocytopenia and evidence of plasma leakage [16]. Hematoconcentration was determined by the hematological analyzer and expressed as % of the volume of whole blood that was made up of red blood cells. Hematocrit increase of over 20% of the values at convalescence phase is considered a common clinical index of plasma leakage and DHF diagnosis. The PADS cohort comprised of both DF and DHF patients recruited at different phases of dengue infection and DENV2 is the predominant DENV type. Based on the anti-DENV IgG and IgM seropositivity or seronegativitiy, immune status of the patients was determined. Anti-DENV IgM seropositivity and IgG seronegativity indicated that 40–52% of DF and 22–41% of DHF are primary cases, and the remaining are secondary cases. 12–16% DF patients and 59–76% DHF patients had platelets <50×10^3^/μL at any point as determined during their daily routine total blood count.

### Hematological, serological and virological analysis

A detailed hematological and virological analysis was performed and included white blood cell count (WBC), red blood cell count (RBC), blood hemoglobin (HGB), hematocrit (HCT), mean corpuscular volume (MCV), mean corpuscular hemoglobin (MCH), mean corpuscular hemoglobin concentration (MCHC), platelet count (PLT), lymphocyte percentage (LYMPH%), lymphocyte count (LYMPH), mixed cell count (MXD), neutrophil percentage (NEUT%), neutrophil count (NEUT), red blood cell distribution width-coefficient of variation (RDW-CV), and quantitation of peripheral viral titers using reverse transcriptase-polymerase chain reaction (RT-PCR) crossover values (C_t_). Dengue viral infection was confirmed by RT-PCR [17], or NS1 detection by Dengue NS1 Ag Strip (Bio-Rad, Marnes-la-Coquette, France) at the Environmental Health Institute, Singapore, or typing by virus isolation and immunofluorescence using DENV type-specific monoclonal antibodies (ATCC: HB46-49). Dengue-immune status (primary or secondary DENV infection) was based on Dengue IgG levels in the acute sera, using a commercially obtained ELISA (PanBio, Brisbane, Australia) according to the manufacturer’s protocol.

### Sample preparation

A volume of 50 μL from each serum sample was thawed at 4°C and serum proteins were precipitated with 200 mL ice-cold methanol, which contained 10 mg/mL 9-fluorenylmethoxycarbonyl-glycine as an internal standard. After vortexing, the mixture was centrifuged at 16,000 rpm for 10 minutes at 4°C and the supernatant was collected and evaporated to dryness in a vacuum evaporator. The dry extracts were then redissolved in 200 μL of 98:2 water/methanol for liquid chromatography-mass spectrometry (LC-MS) analysis. Quality control (QC) samples were prepared by mixing equal amounts of serum samples from all the samples and processed as per other samples. The QC sample was run after each 8 samples to monitor the stability of the system and all samples were randomized.

### Metabolomics analysis by LC-MS

Metabolomics analysis was performed as previously described [14]. Briefly, the supernatant fraction from sample preparation step was analyzed using Agilent 1290 ultrahigh pressure liquid chromatography system (Waldbronn, Germany) equipped with a 6550 QTOF mass detector managed by a MassHunter workstation. The column used for the separation was an Agilent rapid resolution HT Zorbax SB-C18 (2.1×100 mm, 1.8 mm; Agilent Technologies, Santa Clara, CA, USA). The oven temperature was set at 45°C. The gradient elution involved a mobile phase consisting of (A) 0.1% formic acid in water and (B) 0.1% formic acid in methanol. The initial condition was set at 5% B. A 7 min linear gradient to 70% B was applied, followed by a 12 min gradient to 100% B which was held for 3 min, then returned to starting conditions over 0.1 min. Flow rate was set at 0.4 ml/min, and 5 μL of samples was injected. The electrospray ionization mass spectra were acquired in both positive and negative ion mode. Mass data were collected between m/z 100 and 1000 at a rate of two scans per second. The ion spray voltage was set at 4,000 V, and the heated capillary temperature was maintained at 350°C. The drying gas and nebulizer nitrogen gas flow rates were 12.0 L/min and 50 psi, respectively. Two reference masses were continuously infused to the system to allow constant mass correction during the run: m/z 121.0509 (C_5_H_4_N_4_) and m/z 922.0098 (C_18_H_18_O_6_N_3_P_3_F_24_).

### Data analysis

Raw spectrometric data in untargeted metabolomics were analyzed by MassHunter Qualitative Analysis software (Agilent Technologies, US) and the molecular features characterized by retention time (RT), chromatographic peak intensity and accurate mass, were obtained by using the Molecular Feature Extractor algorithm. The features were then analyzed by MassHunter Mass Profiler Professional software (Agilent Technologies, US). Only features with an intensity ≥ 20,000 counts (approximately three times the limit of detection of our LC-MS instrument), and found in at least 80% of the samples at the same sampling time point signal were kept for further processing. Next, a tolerance window of 0.15 min and 2 mDa was used for alignment of RT and *m/z* values, and the data normalized to spiked 9-fluorenylmethoxycarbonyl-glycine internal standard. Raw spectrometric data in targeted metabolomics were processed using MassHunter Workstation Quantitative Analysis software (Agilent Technologies, US).

For statistical analysis, nonparametric Mann–Whitney Test with Benjamini-Hochberg Multiple Testing Correction was employed, and statistical significance was set at *p*<0.05. For multivariate data analysis using principle component analysis (PCA) or Orthogonal projections to latent structures discriminant analysis (OPLS-DA), data were normalized by median-centering and dividing by standard deviation. PCA and OPLS-DA were performed using the software package SIMCA-P 13.0 version (Umetrics AB, Umea, Sweden). Metabolites with Variable Importance in the Projection (VIP) values>1 were considered to be influential for the separation of samples in OPLS-DA analysis. In addition, the fold change (FC) analysis was also performed to further filter the features and only those features with FC > 1.5 were selected as potential significantly altered metabolites. The hierarchical cluster analysis (HCA), a cluster analysis method which seeks to build a hierarchy of clusters, was performed using MeV4.0.

### Compound identification

The structure identification of the differentially expressed metabolites was based on our published work [18]. Briefly, the elemental compositions of the metabolites were first calculated based on the exact mass, the nitrogen rule and the isotope pattern by Masshunter software from Agilent. Then, the elemental composition and exact mass were used for open source database searching with a cutoff value of 10 parts per million (ppm), including LIPIDMAPS (http://www.lipidmaps.org/), HMDB (http://www.hmdb.ca/), METLIN (http://metlin.scripps.edu/) and MassBank (http://www.massbank.jp/). Next, MS/MS experiments were performed to obtain structural information via the interpretation of the fragmentation pattern of the metabolite. The MS/MS spectra of possible metabolite candidates in the databases were also searched and compared. Finally, the metabolites were confirmed by comparison with the standards where commercially available. The metabolites are listed according to the minimum reporting standards for chemical analysis in metabolomics recommended by Metabolomics Standard Initiative (MSI) [19,20]. Briefly, a four-level system ranging from Level 1 (identified metabolites, e.g. based upon the co-characterization with reference standards) via Levels 2 (putatively annotated compounds, e.g. without chemical reference standards, based upon physicochemical properties and/or spectral similarity with public/commercial spectral libraries) and 3 (putatively characterized compound classes, e.g. based upon characteristic physicochemical properties of a chemical class of compounds, or be spectral similarity to known compounds of a chemical class) to Level 4 (unidentified or unclassified metabolites which can still be differentiated based on spectrum data). For metabolic pathway analysis, MetaboAnalyst was used to identify relevant pathways [21].

## Results

### Serum metabolic profiles of DF and DHF patients at critical and recovery phase

We characterized serum metabolome changes between DF (*n* = 25) and DHF (*n* = 27) patients at both the critical phase and recovery phase of infection using LC-MS. We first evaluated the stability and reproducibility of the LC-MS method by performing PCA on all the samples including the 6 QC samples [22]. As shown in S1 and S2 Figs, the QC samples are clustered in PCA scores plots of sera (S1 and S2 Figs), indicating good stability and reproducibility of the chromatographic separation of the metabolomics analysis.

### Identification of significantly altered metabolites in DHF patients at critical and recovery phase

**Fig 1:**
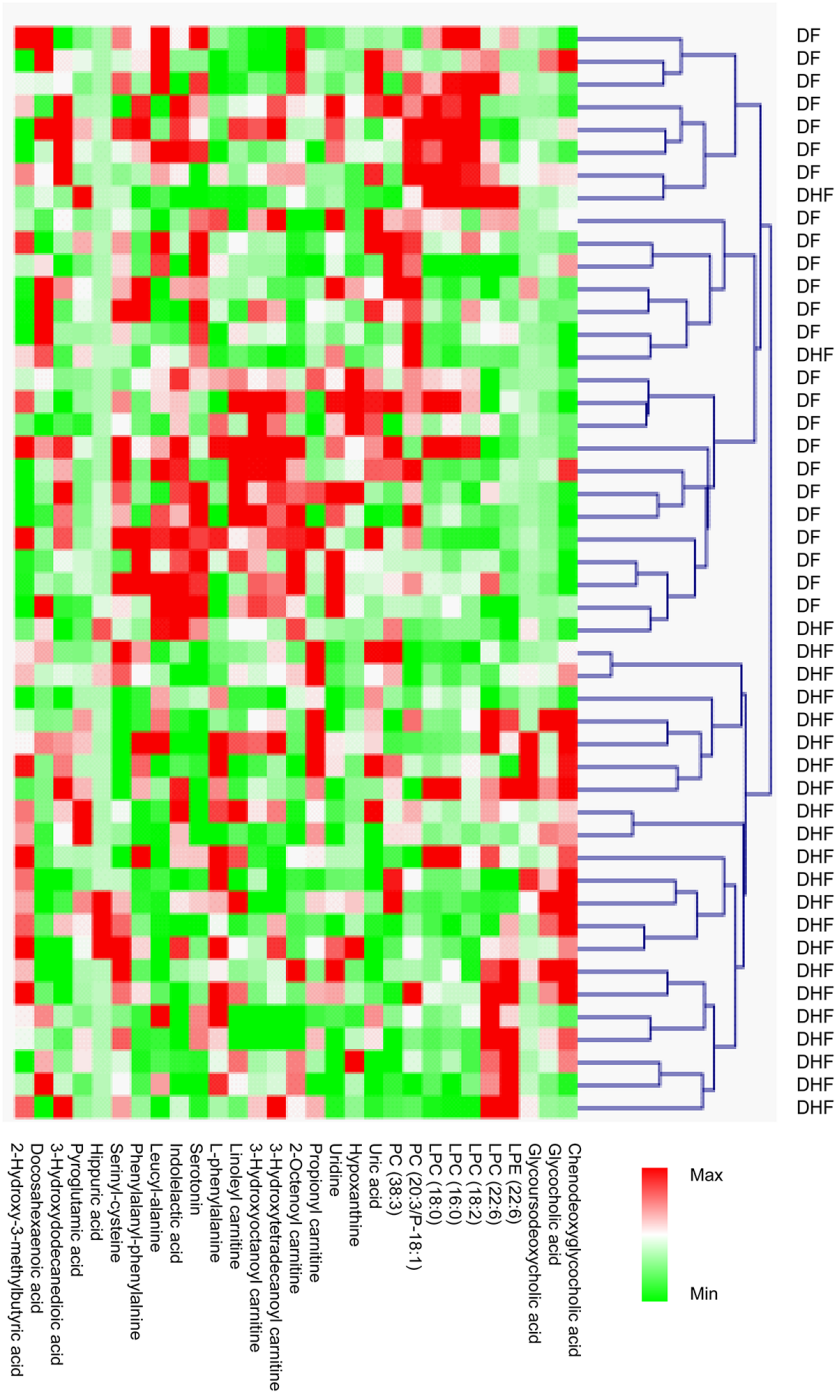
Hierarchical heatmap clustering with identified differentially expressed metabolites segregates critical phase dengue fever (DF) and dengue hemorrhagic fever (DHF) patients. Each column shows ion intensity for a specific metabolite after mean centering and unit variance scaling of the data. Each row shows the serum metabolic profiles of DF and DHF patients.

**Fig 2:**
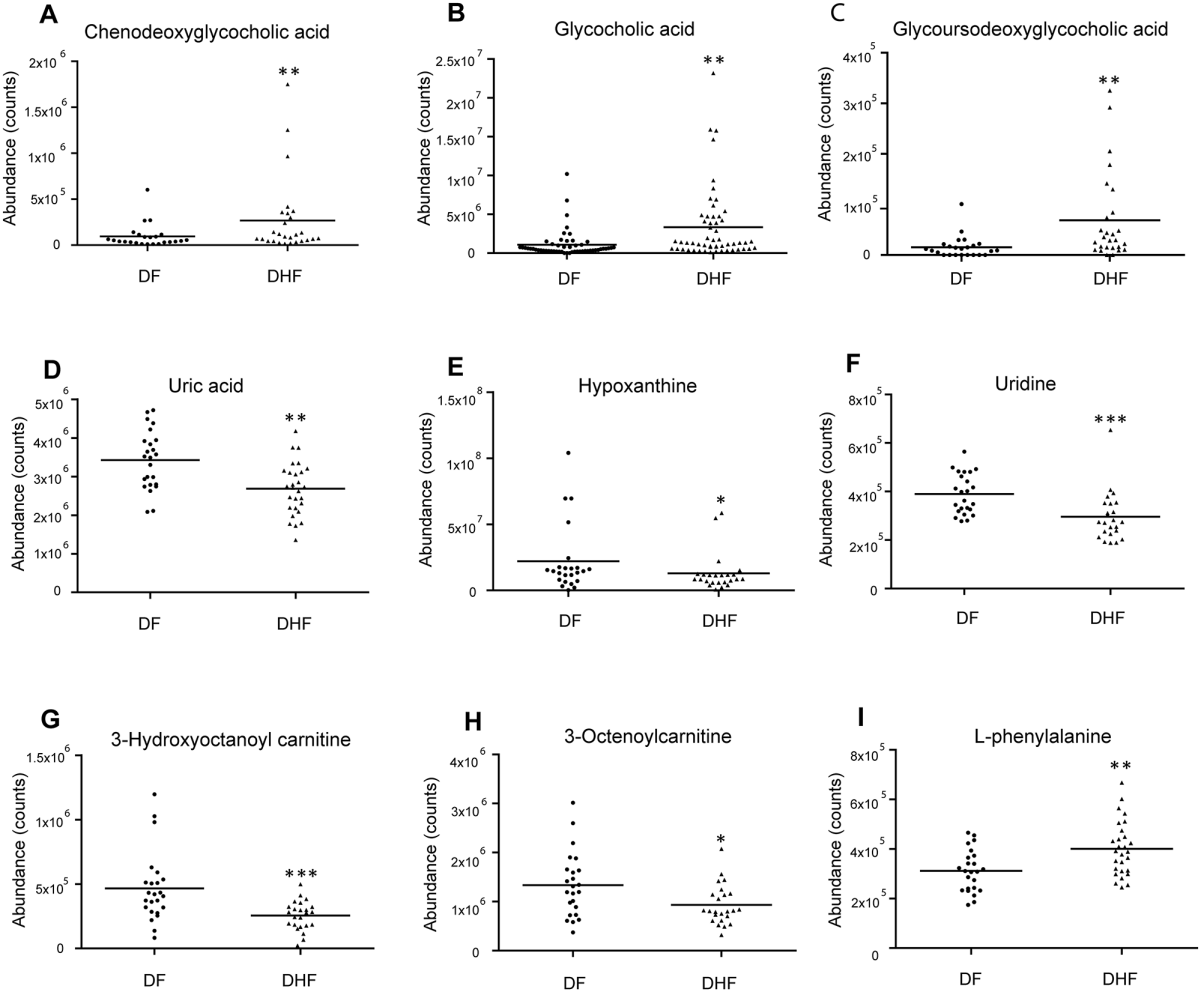
Box plots of representative differentially expressed metabolites between dengue fever (DF) and dengue hemorrhagic fever (DHF) patients during critical phase. **A.** Chenodeoxyglycocholic acid **B.** Glycocholic acid **C.** Glycoursodeoxycholic acid **D.** Uric acid **E.** Hypoxanthine **F.** Uridine **G.** 3-Hydroxyoctanoyl carnitine **H.** 2-Octenoylcarnitine **I.** L-phenylalanine. Horizontal lines represent median value. * *p*<0.05, ** *p*<0.01, *** *p*<0.001, by Mann-Whitney test. The statistical comparison was with DF levels.

**Fig 3:**
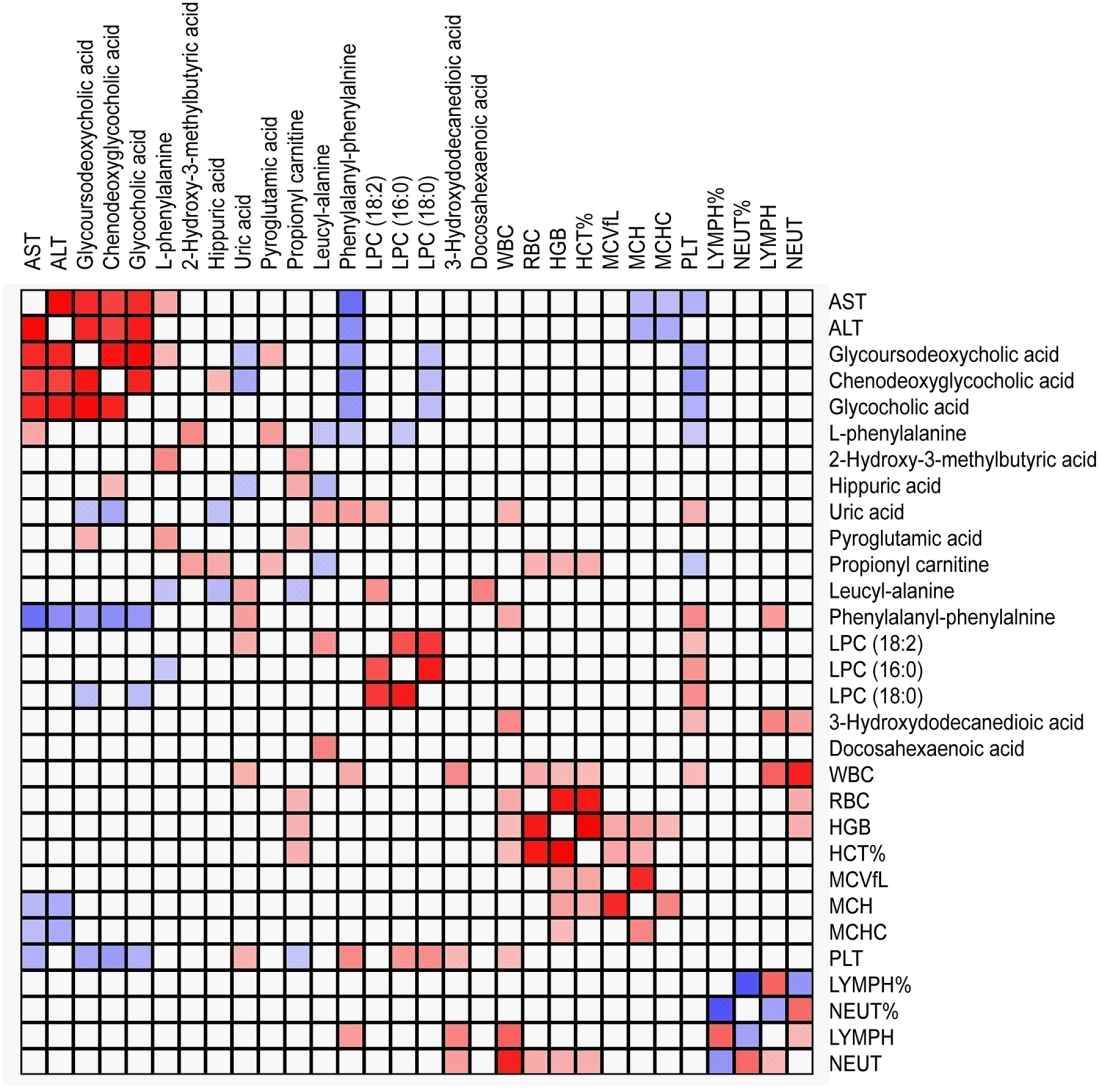
Pearson correlation analysis reveals correlation of bile acids with aspartate transaminase (AST) and alanine transaminase (ALT) levels in the critical phase of infection.

**Fig 4:**
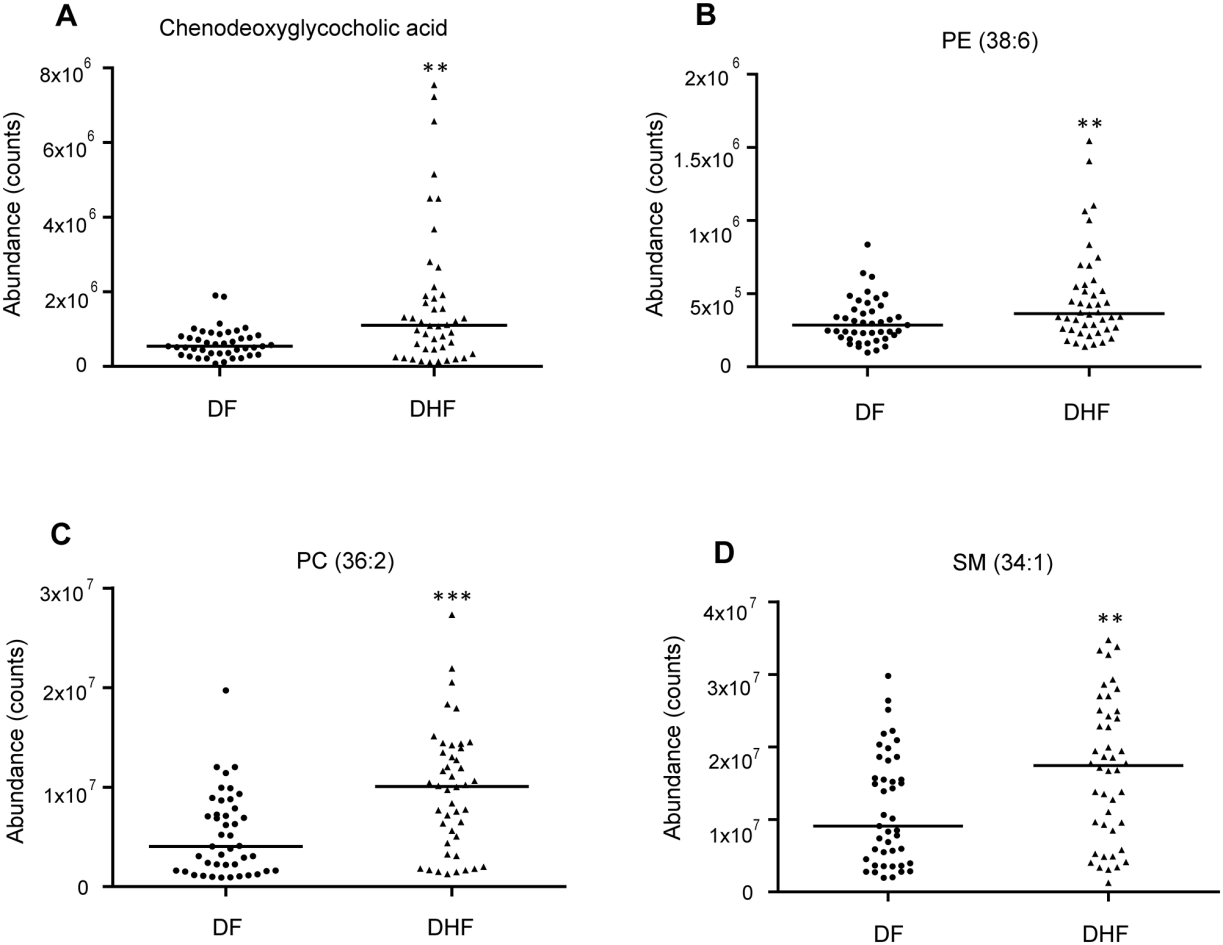
Box plots of representative differentially expressed metabolites between dengue fever (DF) and dengue hemorrhagic fever (DHF) patients during recovery phase. **A.** Chenodeoxyglycocholic acid **B.** PE (38:6) **C.** PC (36:2) **D.** SM (34:1). Horizontal lines represent median value. * *p*<0.05, ** *p*<0.01, *** *p*<0.001, by Mann-Whitney test. The statistical comparison was with DF levels.

In the critical phase, a total of 29 MSI Levels 1 and 2 metabolites, were significantly expressed between DHF and DF patients, were identified. HCA could successively merge similar groups of objects and the objects are joined together in a hierarchical fashion from the closest, that is most similar, to the furthest apart, that is the most different. HCA based on the metabolome profile could segregate DHF and DF patients in the critical phase (Fig 1), where all but two DHF patients were classified together with DF. These differentially expressed metabolites belonged to classes such as bile acid, acylcarnitine, phosphatidylcholine (PC), lysophosphatidylcholine (LysoPC), amino acid and derivative, dipeptide, purine and pyrimidine (Table 1). Compared to DF patients, 10 of the 29 differentially expressed metabolites were increased in DHF patients. Among them, the levels of the bile acids showed about 5 times higher in DHF patients than in DF patients (Fig 2). Furthermore, these bile acids demonstrated positive correlations to AST and ALT levels, indicating they may be associated with liver dysfunction in DHF (Fig 3). The other 19 differentially expressed metabolites, including purines and pyrimidines, most of the acylcarnitines and lipids, were decreased in DHF patients. In the recovery phase, 8 altered MSI Levels 1 and 2 metabolites between DF and DHF patients were structurally identified including bile acid, phospholipids, amino acid, dipeptide, and fatty acid (Table 2). Among these differentially expressed metabolites, the level of the bile acid, chenodeoxyglycocholic acid, was still about 3 times higher in DHF patients than in DF patients, indicating prolonged effects of DENV infection on DHF patients (Fig 4).

**Table 1:** Identified differentially expressed metabolites between dengue fever (DF) and dengue hemorrhagic fever (DHF) patients in the critical phase of infection.

HMDB	Accurate mass	Metabolite	Chemical formula	*p* value	Fold change (DHF/DF)	Pathway
HMDB00289	168.0288	Uric acid*	C_5_H_4_N_4_O_3_	0.0005	0.66	Purine metabolism
HMDB00157	136.0385	Hypoxanthine*	C_5_H_4_N_4_O	0.01	0.52	Purine metabolism
HMDB00296	244.0695	Uridine*	C_9_H_12_N_2_O_6_	0.0003	0.70	Pyrimidine metabolism
HMDB06898	449.3149	Chenodeoxyglycocholic acid	C_26_H_43_NO_5_	0.039	4.63	Bile acid biosynthesis
HMDB00708	449.3142	Glycoursodeoxycholic acid	C_26_H_43_NO_5_	0.0177	5.96	Bile acid biosynthesis
HMDB00138	465.3084	Glycocholic acid	C_26_H_43_NO_6_	0.0357	6.23	Bile acid biosynthesis
HMDB00824	217.1318	Propionylcarnitine	C_10_H_19_NO_4_	0.0073	1.56	fatty acid β-oxidation
HMDB13324	285.194	2-Octenoylcarnitine*	C_15_H_27_NO_4_	0.021	0.70	fatty acid β-oxidation
HMDB61640	387.2984	3-hydroxytetradecanoyl carnitine	C_21_H_41_NO_5_	0.023	0.70	fatty acid β-oxidation
HMDB61634	303.2045	3-hydroxyoctanoyl carnitine	C_15_H_29_NO_5_	0.0007	0.55	fatty acid β-oxidation
HMDB06461	423.3348	Linoleyl carnitine*	C_25_H_45_NO_4_	0.045	0.71	fatty acid β-oxidation
HMDB00259	176.0928	Serotonin*	C_10_H_12_N_2_O	0.0001	0.42	Tryptophan metabolism
HMDB00671	205.0739	Indolelactic acid*	C_11_H_11_NO_3_	0.01	0.56	Tryptophan metabolism
HMDB00159	165.0795	L-Phenylalanine*	C_9_H_11_NO_2_	0.0019	1.59	phenylalanine metabolism
HMDB00714	179.0591	Hippuric acid	C_9_H_9_NO_3_	0.0397	3.59	phenylalanine metabolism
HMDB28922	202.1318	Leucyl-Alanine	C_9_H_18_N_2_O_3_	0.0047	0.63	Dipeptide
HMDB13302	312.1475	Phenylalanylphenylalanine	C_18_H_20_N_2_O_3_	0.0374	0.50	Dipeptide
HMDB29036	208.0517	Serinyl-Cysteine	C_6_H_12_N_2_O_4_S	0.03	0.70	Dipeptide
HMDB10386	519.3316	LysoPC(18:2)	C_26_H_50_NO_7_P	0.0201	0.59	Glycerophospholipid
HMDB10382	495.3321	LysoPC(16:0)	C_24_H_50_NO_7_P	0.0241	0.66	Glycerophospholipid
HMDB10384	523.3635	LysoPC(18:0)	C_26_H_54_NO_7_P	0.045	0.64	Glycerophospholipid
HMDB11526	525.2855	LysoPE(22:6)	C_27_H_44_NO_7_P	0.018	1.56	Glycerophospholipid
HMDB10404	567.3324	LysoPC(22:6	C_30_H_50_NO_7_P	0.025	1.45	Glycerophospholipid
HMDB08392	793.5985	PC(P38:4)	C_46_H_84_NO_7_P	0.003	0.72	Glycerophospholipid
HMDB08020	811.6091	PC(38:3)	C_46_H_86_NO_8_P	0.045	0.71	Glycerophospholipid
HMDB02183	328.2376	Docosahexaenoic acid*	C_22_H_32_O_2_	0.0457	0.58	fatty acid
HMDB00413	246.1467	3-Hydroxydodecanedioic acid	C_12_H_22_O_5_	0.0402	0.54	Organic acid
HMDB01987	118.063	2-Hydroxy-3-methylbutyric acid	C_5_H_10_O_3_	0.006	1.42	Organic acid
HMDB00267	129.0424	Pyroglutamic acid*	C_5_H_7_NO_3_	0.0379	1.51	glutathion metabolism

* Verified with authentic standards

**Table 2:** Identified differentially expressed metabolites between DF and DHF patients in the recovery phase of dengue infection.

HMDB	Accurate mass	Metabolite	Chemical formula	*p* value	Fold change (DHF/DF)	Pathway
HMDB06898	449.3141	Chenodeoxyglycocholic acid	C_26_H_43_NO_5_	0.0095	2.96	Bile acid biosynthesis
HMDB00910	214.1932	Tridecanoic acid	C_13_H_26_O_2_	0.006	0.73	fatty acid
HMDB13464	702.5675	SM(d34:1)	C_39_H_79_N_2_O_6_P	0.0097	1.54	sphingolipid
HMDB08946	763.5152	PE(38:6)	C_43_H_74_NO_8_P	0.0158	1.68	Glycerophospholipid
HMDB00593	785.5934	PC(36:2)	C_44_H_84_NO_8_P	0.0006	1.78	Glycerophospholipid
HMDB00991	159.1259	DL-2-Aminooctanoic acid	C_8_H_17_NO_2_	0.0438	0.70	amino acid
HMDB28757	246.1215	Aspartyl-Leucine	C_10_H_18_N_2_O_5_	0.0183	1.70	Dipeptide
HMDB29118	280.1423	Tyrosyl-Valine	C_14_H_20_N_2_O_4_	0.002	0.68	Dipeptide

To assess their potential in differentiating DF and DHF, Receiver Operating Curve analyses were performed for the differentially expressed metabolites in the critical phase. Several metabolites showed good performing Area Under Curve (AUC), including serotonin (AUC = 0.85, 95% C.I. 0.75–0.95, *p*<0.0001), uridine (AUC = 0.81, 95% C.I. 0.68–0.93, *p* = 0.0002), glycoursodeoxycholic acid (AUC = 0.77, 95% C.I. 0.65–0.90, *p* = 0.0007), uric acid (AUC = 0.76, 95% C.I. 0.63–0.89, *p* = 0.001) (S3 Fig).

We have previously identified more than 20 differentially expressed metabolites between DF and DHF in the early febrile phase of dengue. Surprisingly, a distinct set of differentially expressed metabolites between DF and DHF were found in critical phase as compared to the early febrile phase. There are only two common metabolites between these two phases and they are serotonin and a dipeptide.—We have shown previously through a stable-isotope dilution mass spectrometry method that serotonin was significantly changed among DHF patients as compared to DF patients in the febrile phase, and serotonin remained significantly altered among the DHF patients in the critical phase. This finding demonstrated the high reliability and confidence of our methodologies and results in our previous and current untargeted analysis approach. In addition, for the three bile acids which were significantly increased among DHF patients as compared to DF patients in the critical phase, their levels was also higher among DHF patients in the febrile phase, but it was not statistically significant. On the other hand, a few fatty acid amides were significantly lower among DHF patients as compared to DF patients in the febrile phase, and their levels became similar between DF and DHF patients in the critical phase.

We used MetaboAnalyst, a pathway analysis tool, to determine the underlying biochemical pathways revealed by the identified metabolites. The perturbed metabolic pathways in DHF in the critical phase included bile acid biosynthesis, fatty acid *β*-oxidation, purine and pyrimidine metabolism, phospholipid catabolism, tryptophan and phenylalanine metabolism. (S4 Fig) In the recovery phase, the bile acid biosynthesis and phospholipid catabolism pathways were still altered, while other metabolic pathways were no longer disturbed in DHF patients (S5 Fig).

## Discussion

Dengue is a very dynamic disease, and the symptoms of DHF usually do not occur until the critical phase of infection. The critical phase is known as the danger period of dengue because a small proportion of patients may undergo sudden deterioration over 24–48 h. If the patient recovers, there are no sequelae in uncomplicated dengue, symptoms resolve and clinical parameters normalize. The mechanisms that contribute to the transition from DF to DHF are not well understood, although the pathological differences between DF and DHF suggest differential virus-host interactions in the susceptibility to the disease.

In our previous study a systematic characterization of serum metabolome at early febrile phase (< 96 hours post onset of fever) was reported from adult DF/DHF patients enrolled from a prospective dengue study [14]. In the present study, metabolomics investigation was conducted on the same patient cohort at the critical phase (day 5–7 post onset of fever) and recovery phase (day 21–28 post onset of fever) to map the serum metabolome and identify metabolic pathways linked to dengue progression. Interestingly, the majority of identified differentially expressed metabolites between DF and DHF in the critical phase were different from the ones identified in the early febrile phase, showing that metabolomics study could capture the specific molecular changes in DF/DHF patients at different phases of infection. In the recovery phase, the levels of a number of lipids were still significantly altered in DHF patients, indicating prolonged effects of DENV infection on DHF patients. Our results could help to identify metabolic pathways linked to dengue progression and understand the mechanisms of DHF pathogenesis.

We have previously found that the levels of bile acids are higher in DF patients than in healthy controls at febrile and critical phases of infection [13]. In this study, we further showed that DHF patients had even higher levels of bile acids than DF patients at both critical and recovery phases of infection. Bile acids, which are formed in the liver as the end products of cholesterol metabolism, can not only facilitate hepatobiliary secretion of endogenous metabolites and nutrient absorption in the intestine, but also play important roles in regulating glucose and lipid metabolism through bile acid receptors [23]. When present in abnormally high levels, bile acids are highly toxic to cells, and impaired bile acid homeostasis could contribute to the pathogenesis of liver and intestinal diseases [24]. Many studies have implicated that DENV has adverse effect on liver functions in dengue patients and dengue infection is believed to be an important cause of acute viral hepatitis in endemic countries [25,26]. The hepatocytes and Kupffer cells in the liver could be the targets for DENV replication and a number of liver hepatic histological changes have been observed in dengue patients [26–28]. Accumulated bile acids in the hepatocytes could result in mitochondrial damage and may eventually lead to apoptosis or necrosis [29]. It has been shown that DHF patients had a higher risk of developing acute liver failure (*p* < 0.001) and liver pathology has been viewed as one of the hallmarks of DHF [2, 30]. In the clinic, liver enlargement and elevated transaminases have been used as the clinical features of hepatic histological changes in dengue patients [31], and we indeed found in this study that AST and ALT levels were significantly higher in DHF than in DF. Put together, elevated bile acids among DHF patients and their positive correlations to AST and ALT levels indicated that these bile acids may play an important role in liver pathology in DHF patients. Furthermore, a recent phase II clinical trial showed that INT-747, a potent agonist of bile acid farnesoid X receptor, could improve histological activity and reduce fibrosis in adult patients with non-alcoholic steatohepatitis [32], indicating bile acid receptors may be a potential therapeutic target for liver disease management in DHF.

In this study, decreased uric acid was observed in DHF patients compared to DF patients at the critical phase. Uric acid is an end product of purine catabolism, and numerous studies have shown that uric acid is a major antioxidant in the blood and can help to protect against free-radical oxidative damage [33,34]. It is reported that the total antioxidant capacity in the blood was positively correlated with uric acid concentration, and low circulating levels of uric acid have been associated with disease conditions with a significant redox imbalance due to increased production of oxidant species and decreased capacity of endogenous antioxidant defenses [35,36]. Indeed, increased ROS and NOS have been reported in DHF patients [37]. Furthermore, we have previously found higher levels of phenylalaninein DF patients than in healthy controls. In this study, when compared to DF patients, a further increase of phenylalanine in DHF patients was observed. We have proposed that the accumulation of phenylalanine was due to elevated oxidative stress status in dengue patients where superoxide and peroxynitrite increase oxidation of tetrahydrobiopterin (BH4), thereby depleting the available BH4 pool [38]. BH4 is a co-factor for phenylalanine (4)-hydroxylase (PHA), an enzyme required for converting phenylalanine to tyrosine. Thus, higher level of phenylalanine also indicates increased oxidative stress in DHF patients. Together, decreased uric acid could result in impaired ability to scavenge the ROS and NOS in DHF patients. It had been shown that systemic uric acid administration could increase serum antioxidant capacity in healthy volunteers [39], which might be a potential intervention strategy of the condition of DHF.

In our previous studies, we have shown that anti-inflammatory responses played an important role in modulating pro-inflammatory processes to prevent the development of pathologies by excessive or prolonged inflammation in DF patients, and higher levels of Docosahexaenoic acid (DHA) were found in DF patients than in healthy controls [13]. In current study, a decrease of DHA in DHF patients was observed when compared to DF patients. DHA, an omega-3 polyunsaturated fatty acid, is known for the anti-inflammatory activity by suppressing the production of pro-inflammatory cytokines [40], and the lower level of DHA could indicate impaired anti-inflammatory functions in DHF patients compared to DF patients. We have also found earlier that the levels of many acylcarnitines, essential intermediates of fatty acid *β*-oxidation, were higher in DF patients than in healthy controls, suggesting disturbed energy metabolism in DF patients [13]. Interestingly, when compared to DF patients, decreased levels of acylcarnitine were found in DHF patients in this study, indicating DHF actually induced less disturbance in fatty acid *β*-oxidation than DF.

Voge et al had also conducted mass spectrometry-based serum metabolomics studies on non-dengue (ND) controls, DF and DHF patients and identified 13 metabolites that statistically differentiate DHF, DF and ND groups, including amino acids, lipids and vitamins [12]. Interestingly, most of the 13 differentially expressed metabolites were not found in our current study. This is likely due to the different analytical platforms used in the two studies, hydrophilic interaction liquid chromatography versus RP liquid chromatography, leading to different metabolite coverage. Nevertheless, both studies showed that metabolomics approach could identify metabolites associated with distinct disease outcomes of dengue infection.

In summary, our study identified differentially expressed metabolites and the underlying metabolic pathways linked to DHF in both the critical and recovery phases of dengue infection. These results provide insights into the mechanisms of DHF pathogenesis, and certain altered pathways might serve as therapeutic targets to alleviate DHF.

## Supporting information

S1 Fig PCA scores plots of dengue fever (DF) and dengue hemorrhagic fever (DHF) patients in the critical phase of dengue infection. (PDF)

S2 Fig PCA scores plots of dengue fever (DF) and dengue hemorrhagic fever (DHF) patients in the recovery phase of dengue infection. (PDF)

S3 Fig Receiver Operating Curves of serotonin (A), Uridine (B), Glycoursodeoxycholic acid (C) and Uric acid (D). The sensitivity and specificity refer to distinguishing DF and DHF.
(PDF)

S4 Fig Pathway analysis based on altered metabolites in the critical phase of dengue infection using MetaboAnalyst. (PDF)

S5 Fig Pathway analysis based on altered metabolites in the recovery phase of dengue infection using MetaboAnalyst. (PDF)
