# Supplementary figures and images for: Serum metabolome changes in adult patients with severe dengue in the critical and recovery phases of dengue infection
Source: PLoS Negl Trop Dis. 2018 Jan 24;12(1):e0006217. doi: 10.1371/journal.pntd.0006217 (PMC5798853; doi:10.1371/journal.pntd.0006217)

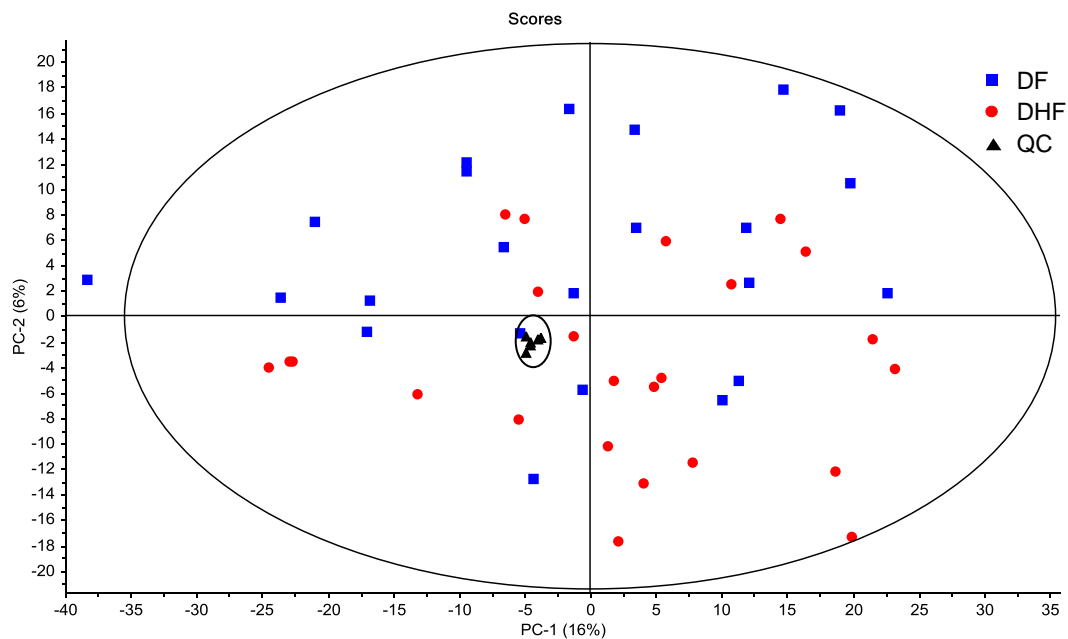

Supplement: S1 Fig — (PDF) [file pntd.0006217.s001.pdf]

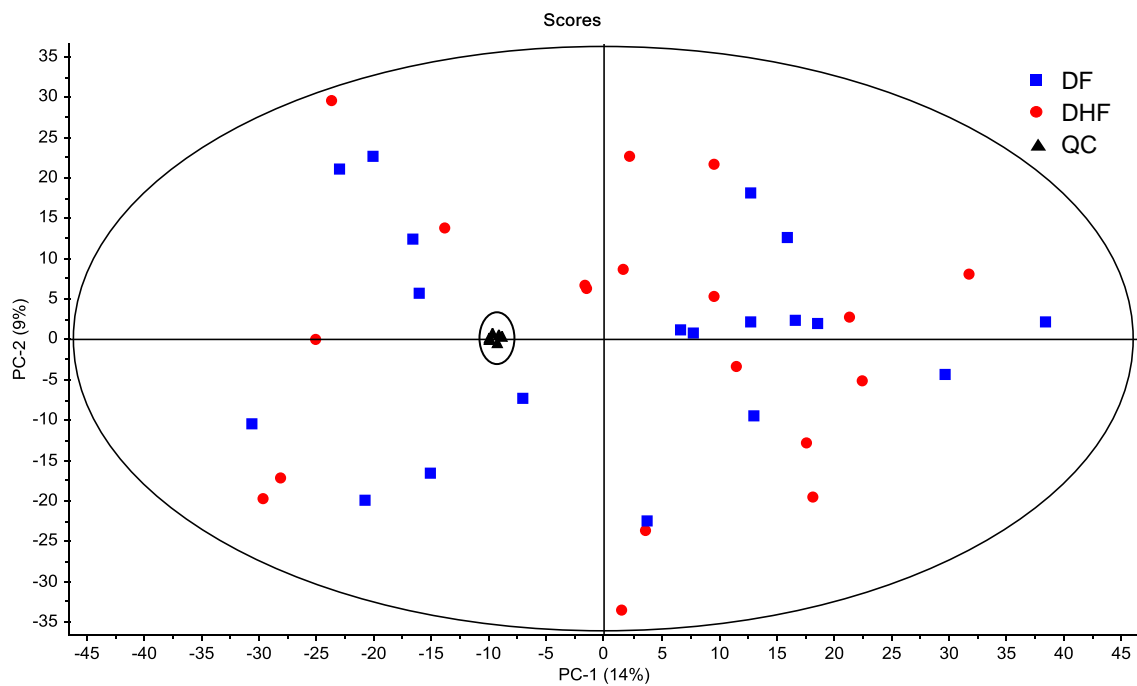

Supplement: S2 Fig — (PDF) [file pntd.0006217.s002.pdf]
