# Supplementary material for: Serum metabolome changes in adult patients with severe dengue in the critical and recovery phases of dengue infection
Source: PLoS Negl Trop Dis. 2018 Jan 24;12(1):e0006217. doi: 10.1371/journal.pntd.0006217 (PMC5798853; doi:10.1371/journal.pntd.0006217)
Supplement: S3 Fig — The sensitivity and specificity refer to distinguishing DF and DHF. (PDF) [file pntd.0006217.s003.pdf]

A

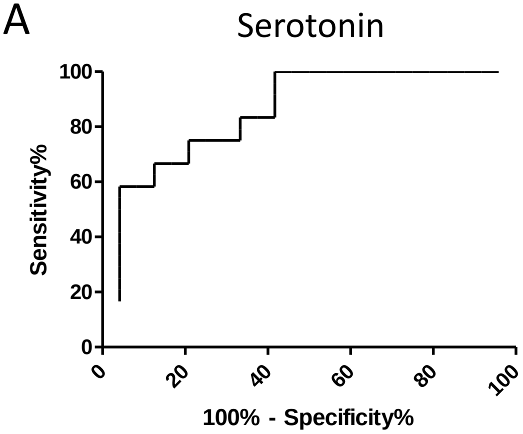

Area	0.85
Std. Error	0.05
95% confidence interval	0.75-0.96
P value	<0.0001

B

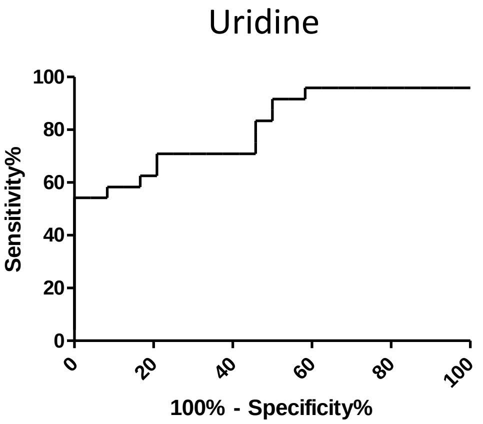

Area	0.81
Std. Error	0.06
95% confidence interval	0.68-0.93
P value	0.0002

C

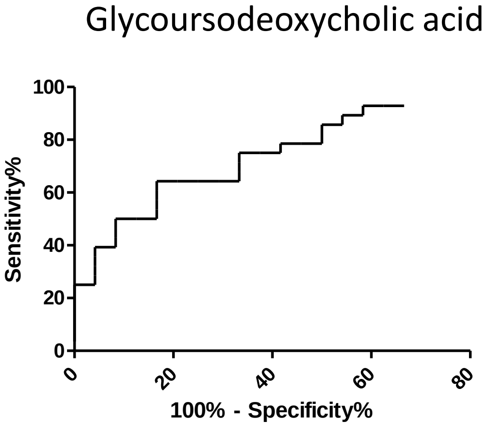

Area	0.77
Std. Error	0.06
95% confidence interval	0.65-0.90
P value	0.0007

D

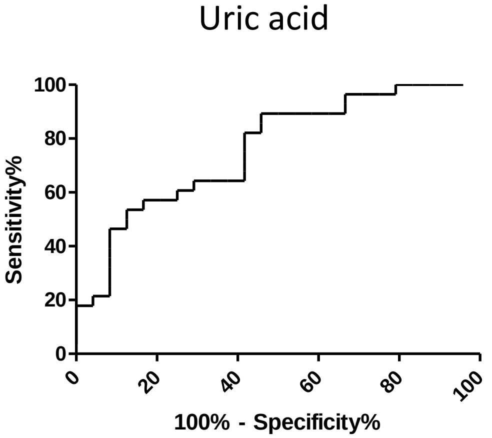

Area	0.76
Std. Error	0.06
95% confidence interval	0.63-0.89
P value	0.001
