# Supplementary material for: Serum metabolome changes in adult patients with severe dengue in the critical and recovery phases of dengue infection
Source: PLoS Negl Trop Dis. 2018 Jan 24;12(1):e0006217. doi: 10.1371/journal.pntd.0006217 (PMC5798853; doi:10.1371/journal.pntd.0006217)
Supplement: S4 Fig — (PDF) [file pntd.0006217.s004.pdf]

	Total	Expected	Hits	Raw p	-log(p)	Holm adjust	FDR	Impact
Phenylalanine metabolism	45	0.15	2	8.91E-03	4.72E+00	7.13E-01	7.13E-01	0.15
Phenylalanine, tyrosine and tryptophan biosynthesis	27	0.09	1	8.64E-02	2.45E+00	1.00E+00	1.00E+00	0.00
Glutathione metabolism	38	0.13	1	1.20E-01	2.12E+00	1.00E+00	1.00E+00	0.00
Nitrogen metabolism	39	0.13	1	1.23E-01	2.10E+00	1.00E+00	1.00E+00	0.00
Primary bile acid biosynthesis	47	0.16	1	1.46E-01	1.92E+00	1.00E+00	1.00E+00	0.01
Aminoacyl-tRNA biosynthesis	75	0.25	1	2.24E-01	1.50E+00	1.00E+00	1.00E+00	0.00
Tryptophan metabolism	79	0.26	1	2.35E-01	1.45E+00	1.00E+00	1.00E+00	0.06
Purine metabolism	92	0.31	1	2.68E-01	1.32E+00	1.00E+00	1.00E+00	0.01
