# Supplementary material for: Serum metabolome changes in adult patients with severe dengue in the critical and recovery phases of dengue infection
Source: PLoS Negl Trop Dis. 2018 Jan 24;12(1):e0006217. doi: 10.1371/journal.pntd.0006217 (PMC5798853; doi:10.1371/journal.pntd.0006217)
Supplement: S5 Fig — (PDF) [file pntd.0006217.s005.pdf]

	Total	Expected	Hits	Raw p	-log(p)	Holm adjust	FDR	Impact
Glycerophospholipid metabolism	39	0.08	2	2.48E-03	6.00E+00	1.98E-01	1.98E-01	0.23
Glycosylphosphatidylinositol(GPI)-anchor biosynthesis	14	0.03	1	2.88E-02	3.55E+00	1.00E+00	8.21E-01	0.04
Linoleic acid metabolism	15	0.03	1	3.08E-02	3.48E+00	1.00E+00	8.21E-01	0.00
Sphingolipid metabolism	25	0.05	1	5.09E-02	2.98E+00	1.00E+00	9.42E-01	0.01
alpha-Linolenic acid metabolism	29	0.06	1	5.89E-02	2.83E+00	1.00E+00	9.42E-01	0.00
Arachidonic acid metabolism	62	0.13	1	1.22E-01	2.10E+00	1.00E+00	1.00E+00	0.00
